# Sprague Dawley rats from different vendors vary in the modulation of prepulse inhibition of startle (PPI) by dopamine, acetylcholine, and glutamate drugs

**DOI:** 10.1007/s00213-023-06444-1

**Published:** 2023-08-14

**Authors:** S.B. Caine, S. Plant, K. Furbish, M. Yerton, E. Smaragdi, B. Niclou, J.M. Lorusso, J.Y. Chang, C. Bitter, A. Basu, S. Miller, C.-Y. Huang, R. Komson, D. Liu, S. Behar, M. Thomsen

**Affiliations:** 1 Neuroscience and Behavioral Pharmacology Laboratory, Department of Psychiatry, McLean Hospital/Harvard Medical School, Belmont, MA USA; 2 Laboratory of Neuropsychiatry, Psychiatric Centre Copenhagen, Mental Health Services in the Capital Region of Denmark, Forskningsenheder, Hovedvejen 17, 1. sal, 2000 Frederiksberg, Copenhagen, Denmark; 3 Department of Neuroscience, Faculty of Health and Medical Sciences, University of Copenhagen, Copenhagen, Denmark

**Keywords:** Prepulse inhibition, Startle response, Sprague Dawley rats, PPI, Dopamine, Acetylcholine, Glutamate, Serotonin, Startle amplitude

## Abstract

**Rationale:**

Rodent vendors are often utilized interchangeably, assuming that the phenotype of a given strain remains standardized between colonies. Several studies, however, have found significant behavioral and physiological differences between Sprague Dawley (SD) rats from separate vendors. Prepulse inhibition of startle (PPI), a form of sensorimotor gating in which a low-intensity leading stimulus reduces the startle response to a subsequent stimulus, may also vary by vendor. Differences in PPI *between* rat strains are well known, but divergence between colonies *within* the SD strain lacks thorough examination.

**Objectives:**

We explored intrastrain variation in PPI by testing SD rats from two vendors: Envigo and Charles River (CR).

**Methods:**

We selected drugs acting on four major neurotransmitter systems that have been repeatedly shown to modulate PPI: dopamine (apomorphine; 0.5, 1.5, 3.0 mg/kg), acetylcholine (scopolamine; 0.1, 0.5, 1.0 mg/kg), glutamate (dizocilpine; 0.5, 1.5, 2.5 mg/kg), and serotonin (2,5-Dimethoxy-4-iodoamphetamine, DOI; 0.25, 0.5, 1.0 mg/kg). We determined PPI and startle amplitude for each drug in male and female Envigo and CR SD rats.

**Results:**

SD rats from Envigo showed dose-dependent decreases in PPI after apomorphine, scopolamine, or dizocilpine administration, without significant effects on startle amplitude. SD rats from CR were less sensitive to modulation of PPI and/or more sensitive to modulation of startle amplitude, across the three drugs.

**Conclusions:**

SD rats showed vendor differences in sensitivity to pharmacological modulation of PPI and startle. We encourage researchers to sample rats from separate vendors before experimentation to identify the most suited source of subjects for their specific endpoints.

**Supplementary Information:**

The online version contains supplementary material available at 10.1007/s00213-023-06444-1.

## Introduction

Rats (*Rattus norvegicus*) are the second-most widely used laboratory animal species after mice, and Sprague Dawley (SD) is one of the most widely employed strains of rat in biomedical research (Krinke et al. 2000). SD and other lines such as Long-Evans and Wistar are available from an array of biotechnology companies, including Envigo (formerly known as Harlan), Charles River Laboratories (CR), and Taconic Biosciences. Researchers have sometimes utilized these vendors interchangeably under the assumption that the SD phenotype remains relatively standardized between isolated colonies. However, vendor differences have been reported in endpoints ranging from basic measures such as bodyweight or blood pressure (Heimlich and Pollock 2014; Pollock and Rekito 1998) to brain monoamine levels or the effects of psychomotor stimulants (Glick et al. 1986; Miller et al. 1968) and operant behaviors (Fitzpatrick et al. 2013).

Vendor colonies of SD rats may also vary in prepulse inhibition of startle (PPI), a form of sensorimotor gating in which a low-intensity leading stimulus reduces the startle response to a subsequent stimulus. This phenomenon is widely used for preclinical evaluation of candidate antipsychotic medications (Geyer et al. 2001). Prior research has found differences in PPI and in modulation of PPI by dopamine receptor agonists between rat strains such as SD, Brown Norway, Wistar, and Long-Evans (Rigdon 1990; Swerdlow et al. 2008a; Swerdlow et al. 2001; Swerdlow et al. 1998; Varty and Higgins 1994). Strain differences in the modulation of PPI were similarly reported for the NMDA glutamate receptor antagonist phencyclidine, suggesting that the strain differences in PPI modulation extend to more neurotransmitter systems than dopamine (Varty and Higgins 1994). Although no significant divergence was seen in dopaminergic modulation of PPI between Harlan SD colonies separated by 11 years of genetic isolation (Swerdlow et al. 2001), SD rats from Harlan (USA) and Bantin-Kingman (UK) did differ (Swerdlow et al. 2000a). Intrastrain differences might also be seen in PPI magnitude and/or in startle amplitude for other classes of psychoactive drugs (abstract by (Rush and Selk 1998)). Finally, very few strain comparisons and no vendor comparisons included female subjects.

The present study seeks to systematically explore intrastrain variation in PPI by analyzing male and female SD rats from two different vendors, CR and Envigo. In a series of studies to investigate potential differences in PPI and startle amplitude, we selected drugs acting on four major neurotransmitter systems that have been repeatedly established across laboratories to modulate PPI (Geyer et al. 2001): dopamine, acetylcholine, glutamate, and serotonin. Specifically, we tested the non-subtype selective dopamine receptor agonist apomorphine, the non-subtype selective competitive muscarinic acetylcholine receptor antagonist scopolamine, the noncompetitive NMDA glutamate antagonist dizocilpine (also known as MK-801), and the serotonin (5-HT) 2A/C agonist 2,5-Dimethoxy-4-iodoamphetamine (DOI). Doses were selected to produce full dose-effect functions up to a plateau or undesirable effects to provide information on differences in potency as well as (maximum) effectiveness of the drugs between experimental groups.

## Materials and methods

### Animals and housing

We tested sixteen experimentally naïve SD rats (8 male, 8 female) from each vendor: Envigo (Indianapolis, IN) and CR (Wilmington, MA). Rats were left to acclimate to the housing facilities at least one week before experiments began. Female rats were ordered in the weight class 125–149 g and male rats in the weight class 150–174 g. The rats were group-housed 2-3/cage, maintained on a nocturnal 12h light-dark schedule in a temperature- and humidity-controlled vivarium, and were fed standard rodent diet (Purina 5001). Females were fed ad libitum, while males were restricted to a daily intake of 24 g after exceeding 330 g in body weight in order to prevent them from becoming unhealthfully obese and uncomfortably large for the 11 cm diameter startle tubes. Tap water was provided ad libitum for both sexes. All procedures were carried out in accordance with the NIH Guide for the Care and Use of Laboratory Animals and the Principles of Laboratory Animal Care and were approved by the McLean Hospital Institutional Animal Care and Use Committee. Drug administrations and behavioral testing were conducted during the dark phase.

### Apparatus and session

Behavioral procedures were standardized as described previously (Caine et al. 1995; Ralph and Caine 2005). In brief, four startle chambers (SR-LAB, San Diego Instruments, San Diego, CA) were used, calibrated to equivalent accelerometer sensitivity and decibel output prior to the study. Startle amplitudes were measured using piezoelectric accelerometers, collecting readings for 100 ms following each stimulus onset; the average movement across this time was used as the startle amplitude for each trial. Each session started with a 5-min acclimation period during which a broadband white background noise (65 dB) was played. Then, the session was composed of four different trial types: a “prepulse” stimulus alone (20 ms duration, 73 dB, i.e., 8 dB above the background noise level), a “pulse” stimulus alone (40 ms, 120 dB, 55 dB above background), a prepulse stimulus followed, after a 100-ms delay onset-to-onset, by a pulse stimulus, or no stimulation (background noise). Initially, five pulse stimuli were presented over the course of 75 s; these trials were omitted from the data analysis because dramatic habituation is expected after initial pulse stimuli (Caine et al. 1995; Geyer et al. 2001; Ralph and Caine 2005; Swerdlow et al. 2008a; Swerdlow et al. 2001). Thereafter, eighteen trials of each noise treatment were presented in a pseudorandom sequence with an inter-trial interval averaging 15 s, for a total session duration of 18 min. Chambers were cleaned with a paper towel and water after each session. Rats from both vendors were tested contemporaneously, with each rat assigned one apparatus, balanced for vendor. Male and female rats were tested on different days, the apparatus was thoroughly cleaned at the end of a test day, and at least one day separated tests of different sexes.

### Drug-modulated PPI

Dose-effect functions were assessed for apomorphine (0, 0.5, 1.5, 3.0 mg/kg), dizocilpine (0, 0.5, 1.5, 2.5 mg/kg), scopolamine (0, 0.1, 0.5, 1.0 mg/kg), and DOI (0, 0.25, 0.5, 1.0 mg/kg). The highest dose of dizocilpine resulted in adverse symptoms in the first few CR females tested (severe diarrhea and splayed posture) and was therefore discontinued in CR females. In addition, DOI was tested in the male rats, but failed to alter PPI up to doses that produced adverse effects (lethargy, splayed posture). DOI was therefore terminated from the study before testing was explored in females, as testing was not deemed ethically sound in the face of high risk of adverse effects and low likelihood of useful effect findings. The preliminary data obtained in the males are shown in Supplemental Figure S1 online. Drug doses including vehicle were tested according to a Latin square design within-subjects. Rats were allowed at least 3 days between tests and at least 1 week between each drug. All drugs were injected subcutaneously (SC) in a volume of 1 ml/kg, 5 min before placing the rat in the test chamber (i.e., 10 min before the first startle stimulus). Drug doses were chosen based on previous studies (Caine et al. 1995; Geyer et al. 2001; Jones and Shannon 2000a; Mansbach and Geyer 1989).

### Data analysis

The two major dependent variables of the study were (1) raw startle amplitudes during the “pulse” stimulus alone trials, averaged across trials in each session, and (2) %PPI averaged across trials in each session, calculated from the startle amplitudes for prepulse + pulse and pulse-alone trials as follows: %PPI = 100 –[(startle response for prepulse + pulse)/(startle response for pulse alone) × 100].

Data were analyzed for each drug separately. Initially, percent PPI and startle amplitude were each analyzed by separate three-way ANOVAs with vendor and sex as between-subjects factors and drug dose as a within-subjects (repeated measures) factor. There were significant sex effects or interactions involving sex in all data sets except for apomorphine PPI modulation (see supplemental Table S1). Therefore, to maintain a uniform analysis across drugs, all data were analyzed and presented as males and females separately as well as sexes combined, i.e., two-way ANOVA with vendor and drug as factors, followed by post hoc Holm-Sidak multiple comparisons test for vendor differences at each dose. Because the highest dose of dizocilpine was omitted in the female CR rats resulting in missing values in the ANOVA design, a mixed effects model was employed for those analyses. Significant drug, vendor, or interaction effects were explored by one-way repeated-measures ANOVA of drug effects in each vendor, followed by Dunnett’s multiple comparisons test of drug doses vs. vehicle. Huynh-Feldt correction for departure from sphericity was applied to repeated-measures analyses including interactions. Findings of *p*<0.05 are described as statistically significant by convention, but “non-significant” *p* values are also reported for transparency.

### Drugs

R-(-)-Apomorphine hydrochloride hemihydrate was diluted in a vehicle of 0.1% ascorbate acid in sterile water; scopolamine hydrobromide, (+)-MK-801 hydrogen malate (dizocilpine), and DOI were dissolved in sterile water. All drugs were purchased from Sigma-Aldrich (St Louis, MO). Doses refer to weights of the drug salts.

## Results

### Pharmacological modulation of PPI

**Fig. 1:**
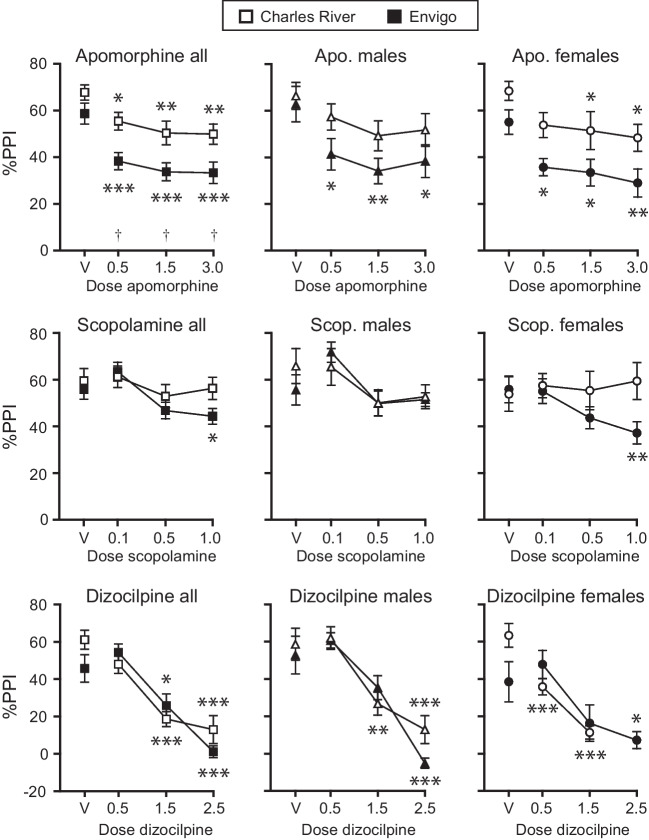
Effects of apomorphine, scopolamine, and dizocilpine on prepulse inhibition. Percent PPI as a function of drug dose. Abscissae: doses of drugs in mg/kg, V denotes vehicle. Ordinates: percent prepulse inhibition of acoustic startle. Open symbols denote Charles River Sprague-Dawley rats and closed symbols denote Envigo Sprague-Dawley rats. *p<0.05, **p<0.01, ***p<0.001 vs. vehicle in each vendor group (Dunnett’s); †*p*<0.05 CR vs. Envigo at each dose (Holm-Sidak)

Figure 1 shows the effects of apomorphine, scopolamine, and dizocilpine on PPI in rats from each vendor.

%PPI was related to both vendor and apomorphine dose. Specifically, %PPI decreased as a function of apomorphine dose in the overall (sexes-combined) analysis (*F*_3,84_=8.3, *p*<0.0001), in the male rats (*F*_3,36_=6.3, *p*=0.002), and in the female rats (*F*_3,42_=9.9, *p*=0.0001). PPI was generally lower in the Envigo rats compared to the CR rats with significant vendor effects as sexes-combined (*F*_1,36_=15, *p*=0.0004) and in the females (*F*_1,22_=12, *p*=0.002), with a near-significant effect in the males (*p*=0.067). The vendor difference reached significance post hoc in the sexes-combined analysis at all apomorphine doses (*p*<0.05) but not vehicle. There were no significant vendor by apomorphine interactions (all *p*>0.6). The effect of apomorphine dose was significant in rats of both CR and Envigo rats overall (*p*=0.003, *p*<0.0001) and in the females (*p*=0.008, *p*=0.01), while only the male Envigo rats showed an effect of apomorphine (*p*=0.007; CR rats *p*=0.22). See Fig. 1 for post hoc significance at individual doses.

%PPI varied as a function of scopolamine dose in the overall analysis [*F*_3,81_=7.4, *p*=0.0002] and in males [*F*_3,36_=7.1, *p*=0.0007], with no significant vendor by scopolamine interaction, while females showed a vendor by scopolamine interaction [*F*_3,39_=3.6, *p*=0.03] and no significant main effect of scopolamine dose. Scopolamine decreased %PPI in the Envigo rats (combined *p*=0.0005, males *p*=0.005, females *p*=0.04) but had no significant effect in the CR rats (*p*=0.07 in the males, otherwise *p*>0.26).

Finally, %PPI decreased as a function of dizocilpine dose in the overall analysis [*F*_3,73_=39, *p*<0.0001], in males [*F*_3,36_=35, *p*<0.0001], and in females [*F*_3,32_=15, *p*<0.0001]. There was a significant vendor by dizocilpine interaction in the females [*F*_2,32_=4.4, *p*=0.03] (sexes-combined analysis, *p*=0.08), which appeared mainly attributable to the vehicle point. The main vendor effect was not significant (*p*≥0.5), and dizocilpine decreased PPI in both CR and Envigo rats in all analyses (*p*=0.01 or lower). In the female CR rats, dizocilpine decreased %PPI to 18.6% at the 1.5 mg/kg dose and was therefore not tested at the 2.5 mg/kg dose.

Although CR rats appeared to have moderately higher baseline PPI than Envigo rats in some tests, this was not consistent. Despite an apparent lower PPI in the Envigo females in the dizocilpine test, analysis of the vehicle tests from each drug revealed no significant effect of vendor, time (repeated tests), or interaction, either combined or in each sex (*p*>0.1, see Supplemental Figure S2A).

### Pharmacological modulation of startle amplitude

**Fig. 2:**
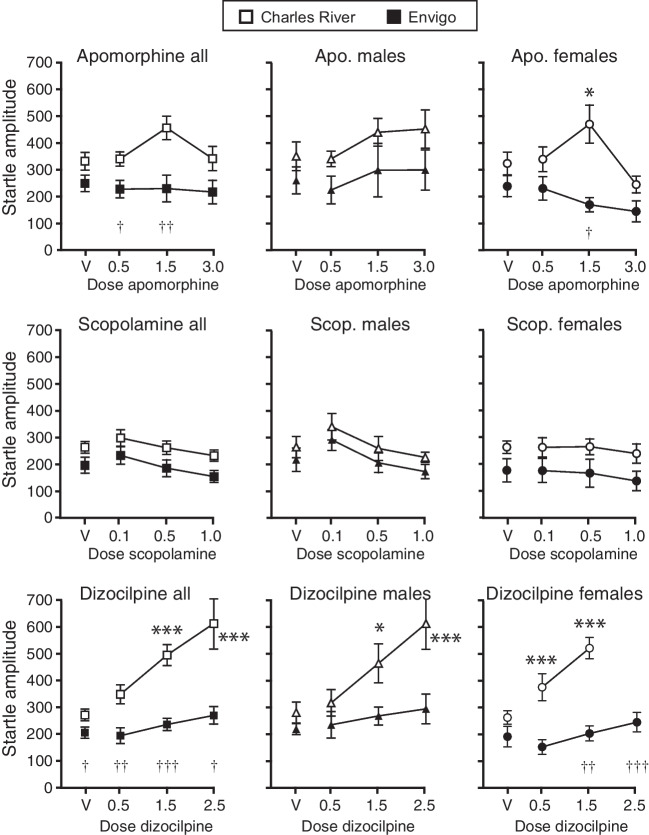
Effects of apomorphine, scopolamine, and dizocilpine on startle amplitude. Startle amplitude during startle (“pulse”) alone trials, as a function of drug dose. Abscissae: doses of drugs in mg/kg, V denotes vehicle. Ordinates: amplitude of acoustic startle (arbitrary units). Open symbols denote Charles River Sprague-Dawley rats and closed symbols denote Envigo Sprague-Dawley rats. *p<0.05, **p<0.01, ***p<0.001 vs. vehicle in each vendor group; †*p*<0.05, ††*p*<0.01, †††*p*<0.001 CR vs. Envigo at each dose (Holm-Sidak)

Figure 2 shows the effects of apomorphine, scopolamine, and dizocilpine on startle amplitude in rats from each vendor. Apomorphine treatment did not produce a significant main effect on startle amplitude in sexes combined, females, or males, although there was a trend in the males (*p*=0.07). However, the CR rats showed higher startle amplitudes than the Envigo rats in the sexes-combined analysis [*F*_1,36_=7.8, *p*=0.008] and in females [*F*_1,22_=8.5, *p*=0.008] (males, *p*=0.12). The female rats showed a trend toward a vendor by apomorphine interaction (*p*=0.07). Specifically, apomorphine increased startle amplitude with a biphasic effect in the female CR rats (main dose effect *p*=0.008, see Fig. 2 for post hoc analyses) and showed a trend toward decreasing startle amplitude in the Envigo females (*p*=0.06). The vendor difference reached significance post hoc in the sexes-combined analysis for 0.5 and 1.5 mg/kg apomorphine and in females at 1.5 mg/kg (see Fig. 2).

Startle amplitude varied moderately by scopolamine dose [*F*_3,81_=5.3, *p*=0.002] and vendor [*F*_1,27_=5.3, *p*=0.03] in the sexes-combined analysis, startle amplitude being again higher in the CR rats, without significant vendor by scopolamine interaction (*p*>0.9). The vendor effect did not reach significance in each sex separately (females *p*=0.08, males *p*=0.2), and the scopolamine effect was significant in males only [*F*_3,36_=4.7, *p*=0.007] (females *p*=0.3), with no vendor by scopolamine interactions (*p*>0.9). The follow-up analyses showed significant scopolamine dose effects in the Envigo rats as sexes combined only (*p*=0.04), but with no dose differing significantly from vehicle levels post hoc.

Startle amplitude showed marked effects of dizocilpine dose, vendor, and vendor by dizocilpine interactions in the overall analysis ([*F*_3,73_=24, *p*<0.0001], [*F*_1,27_=29, *p*<0.0001], [*F*_3,73_=11, *p*=0.0001], respectively), in the males ([*F*_3,36_=8.8, *p*=0.0007], [*F*_1,12_=7.9, *p*=0.02], [*F*_3,36_=3.6, *p*=0.04]), and in the females ([*F*_3,32_=19, *p*<0.0001], [*F*_1,13_=17, *p*=0.001], [*F*_3,32_=20, *p*<0.0001]). Specifically, dizocilpine strongly and dose-dependently increased startle amplitude in CR males (*p*=0.002), females (*p*=0.005), and overall (*p*=0.007; see Fig. 2 for post hoc significance). In contrast, dizocilpine had only modest effects in the Envigo rats (sexes-combined analysis *p*=0.007, females *p*=0.005, males *p*>0.2), which did not reach significance post hoc at individual doses. CR rats had higher startle amplitudes than Envigo rats at all doses including vehicle overall and at the higher doses in the females (see Fig. 2).

CR rats tended to have moderately higher baseline startle amplitude than Envigo rats across tests, and analysis of the vehicle tests from each drug revealed significant effects of vendor [*F*_1,27_=6.4, *p*=0.02], time (i.e., successive tests) [*F*_2,54_=6.6, *p*=0.007], with no time by vendor interaction (*p*=0.5), in the combined analysis (see Supplemental Figure S2B). Post hoc vendor comparisons at each test did not reach significance (Holm-Sidak test). The time effect reflected habituation between the first and subsequent tests, in both vendors. Although the trend was the same in both sexes, effects did not reach significance when analyzed in each sex separately (females *p*=0.06, males *p*=0.20).

## Discussion

Our results indicate that SD rats from Envigo and CR differ in their dose-dependent sensitivity to the PPI-disrupting effects of dopamine receptor agonists, muscarinic cholinergic receptor antagonists, and glutamate NMDA receptor antagonists. The SD rats from Envigo were generally more susceptible to disruption of PPI across the pharmacological treatments, consistent with previous reports in male rats (available only in abstract form) (Hitchcock et al. 1999; Rush and Selk 1998). Specifically, the dopamine receptor agonist apomorphine was more potent and effective at disrupting PPI in Envigo relative to CR rats, particularly in the males. The muscarinic cholinergic antagonist scopolamine was more effective at disrupting PPI in Envigo versus CR SD rats, particularly in the females. The glutamate NMDA receptor antagonist dizocilpine was comparably potent and effective in disrupting PPI in Envigo and CR SD rats, but dizocilpine was profoundly more potent and effective in increasing startle amplitude in CR rats than in Envigo rats. [In the following discussion, when referring to rats from Envigo/Harlan in the literature, we use the vendor name that was in use when the studies were published.]

Apomorphine appeared to modulate PPI with higher efficacy in the Envigo rats generally. The male Envigo rats especially showed a strong PPI decrease at all apomorphine doses tested, while the male CR rats showed no significant effect in the same dose range (without baseline/vehicle difference). It is possible that a higher or lower dose of apomorphine may have affected PPI in the CR rats, but the intermediate dose appeared to be the most effective, suggesting that the dose range selected reached a plateau. In the females, interpretation is less straightforward since baseline (vehicle) PPI was also lower in the Envigo rats relative to the CR rats. Nevertheless, the lowest dose of apomorphine tested significantly decreased PPI in the Envigo, but not CR, female rats, suggesting a higher potency in the female Envigo vs. CR rats. Conversely to its effects on PPI, apomorphine had little effect on startle amplitude, tending to increase this measure in the CR rats but not in the Envigo rats.

Scopolamine decreased PPI significantly only in the female rats from Envigo. It is possible that higher doses may have affected PPI in males and in CR rats. Despite this limitation, we uncovered a clear vendor difference in scopolamine-modulated PPI. It is unclear why we found relatively restricted effects of scopolamine in this investigation; previous studies reported significant PPI disruption by scopolamine, 0.3 and 1 mg/kg, in male SD rats from Harlan, across a range of prepulse intensities, albeit at a lower startle stimulus intensity than the one used here (Jones and Shannon 2000a, 2000b). Using genetically engineered (knockout) mice, we previously found that female mice were more sensitive than male mice to disruption of PPI by muscarinic receptor deletion, although scopolamine decreased PPI in both sexes in the wild-type controls (Thomsen et al. 2010). This suggests the possibility of more general sex differences in cholinergic modulation of PPI, a topic that may deserve further investigation. Scopolamine did not affect startle amplitudes in any groups, consistent with previous research (Jones and Shannon 2000a, 2000b).

Dizocilpine produced the largest decreases in PPI of the drugs tested, with a clear dose-response pattern in rats from both vendors and in both sexes but no clear vendor differences. However, the CR and Envigo rats differed markedly in their sensitivity to dizocilpine in terms of startle amplitude: dizocilpine strongly and dose-dependently increased startle amplitude in the CR rats of both sexes but had little effect in the Envigo rats. If one considers that “the only unambiguous changes in sensorimotor gating are ones that can be demonstrated in the absence of changes in startle magnitude” (Swerdlow et al. 2000b; Swerdlow et al. 2008b), then a rat stock that shows pharmacological modulation of PPI in the absence of effects on startle amplitude is desirable. While dizocilpine consistently decreased PPI in rats in published reports, its effects on startle amplitude at the same doses have been more varied and often amounted to moderate trend-level increases in startle amplitude such as those we observed in the Envigo rats. Dizocilpine (0.3–3 mg/kg) also failed to affect startle amplitude in male mice from five different inbred strains and outbred stocks (Varty et al. 2001). Those earlier reports suggest effects may vary not only between vendors but between breeding locations, as significant increases were found in studies that used rats from Harlan San Diego or from Swedish B&K Universal (Bakshi et al. 1994; Johansson et al. 1995; Varty et al. 1999; Zhang et al. 1997), while no effect or only trends were reported when using rats from Harlan Indianapolis (Keith et al. 1991; Nespor and Tizabi 2008). The present investigation used rats from the Indianapolis location of Envigo. Our results are thus consistent with previous findings but also suggest that results may not generalize between Envigo breeding locations — a finding which has been reported for modulation of startle by apomorphine (Swerdlow et al. 2001), as well as for other measures (see section below).

DOI decreased PPI in male SD rats from Harlan in previous studies (Farid et al. 2000; Sipes and Geyer 1994; Sipes and Geyer 1997). We detected no significant effects of DOI in the present investigation. Possible reasons for the apparent discrepancy include vendor differences (Harlan California through the 1990s vs. Envigo Indianapolis two to three decades later). Experimental parameters may also explain the lack of effect in the present study, in which only one combination of prepulse intensity, pulse intensity, and interstimulus interval was tested. Indeed, the effects of DOI were dependent upon prepulse intensity in previous studies (Farid et al. 2000; Sipes and Geyer 1994), with conditions close to those used in the present investigation not always yielding significant effects of DOI. Like our results, previous studies, using a similar range of DOI doses, found no effect on startle amplitude (Farid et al. 2000; Sipes and Geyer 1994). Finally, we tested DOI after apomorphine, scopolamine, and dizocilpine, and it is possible that repeated testing and/or carry-over effects of repeated drug treatments affected these results.

Comparisons of Harlan (Envigo) SD rats, Long-Evans rats, and their offspring have suggested that the different sensitivities in modulation of PPI by apomorphine were due to genetic rather than environmental factors (Swerdlow et al. 2001; Swerdlow et al. 2004). Genetic variability in male SD rats from several North American CR and Envigo locations was recently investigated in a large genome-wide association study (Gileta et al. 2022), finding that the two vendor populations are highly divergent genetically. Further, a population structure with divergent allele distribution was apparent between locations of the same vendor and even between barrier facilities at each location, especially for Envigo (Gileta et al. 2022). Similarly, in measures of food-rewarded operant behavior, male rats varied as much between colonies of the same vendor as between CR and Harlan (Fitzpatrick et al. 2013). Such detailed genetic mapping may open possibilities for hypothesis generation regarding genetic (or environmental) factors underlying the differences between CR and Envigo rats in the present studies.

PPI after vehicle administration did not differ systematically between the vendors in the present study; PPI appeared stable in the male rats but more variable between tests in the females. The Envigo female rats tended to have lower PPI in the apomorphine and dizocilpine determinations only, perhaps suggesting some carry-over effect of lower PPI after pretreatments that significantly decreased PPI. Alternatively, it is possible that repeated testing and/or between-drugs carry-over effects affected PPI levels in female Envigo rats differentially, a hypothesis that could be verified by repeating the present experiment in separate, drug-naïve, cohorts of rats for each drug. Given the generally comparable levels of baseline PPI, the observed vendor differences in PPI are likely due to differences in sensitivity to the pharmacological manipulations rather than baseline differences. Furthermore, in studies that grouped CR male rats as “low” or “high” baseline PPI, the rats with higher baseline PPI showed greater sensitivity to pharmacological disruption including by apomorphine, scopolamine, and dizocilpine, not lower (Oral and Goktalay 2021). In addition, we observed decreases in PPI without changes in startle amplitude in at least some groups for each drug. Specifically, the decreases in PPI observed in the Envigo rats can reasonably be thought to reflect modulation of sensory gating, because they were not accompanied by changes in startle amplitude. In contrast for the CR rats, interpretation of the decreases in PPI is complicated by the fact that they were almost always accompanied by increases in startle amplitude. While startle amplitude decreased between the first (apomorphine) and second (scopolamine) set of tests, which could affect drug sensitivity, this decrease happened regardless of vendor, and the moderate vendor difference in startle amplitude remained consistent across the study.

In conclusion, our findings on measures of PPI and startle, together with previous studies that indicate intrastrain diversity in this and other behaviors, suggest that SD vendor colonies have undergone genetic drift, leading to significant phenotypic differences. Such findings should encourage researchers to sample rats from several separate vendors prior to large-scale experimentation in order to gauge which stock is most sensitive to their experimental manipulations and endpoint measures. For the specific goal under study here, namely sensitivity to pharmacological manipulation of prepulse inhibition of the startle reflex by dopamine, acetylcholine, and glutamate systems, the male and female SD rats from Envigo appeared better suited than the CR SD rats.

## Supplementary information

ESM 1

## Abbreviations

*CR*: Charles River
*DOI*: 2,5-Dimethoxy-4-iodoamphetamine
*NMDA*: N-methyl-D-aspartate
*PPI*: Prepulse inhibition
*SC*: Subcutaneously
*SD*: Sprague Dawley
