# Supplementary material for: Sprague Dawley rats from different vendors vary in the modulation of prepulse inhibition of startle (PPI) by dopamine, acetylcholine, and glutamate drugs
Source: Psychopharmacology (Berl). 2023 Aug 14;240(9):2005–12. doi: 10.1007/s00213-023-06444-1 (PMC10471717; doi:10.1007/s00213-023-06444-1)
Supplement: Supplementary file 1 — ESM 1 [file 213_2023_6444_MOESM1_ESM.docx]

**Sprague Dawley rats from different vendors vary in prepulse inhibition of startle (PPI)**

Supplemental information

Caine SB, Plant S, Furbish K, Yerton M, Smaragdi E, Niclou B, Lorusso JM, Chang JY, Bitter C, Basu A, Miller S, Huang C-Y, Komson R, Liu D, Behar S, Thomsen M

**Supplemental Figure S1. Effects of DOI on PPI**

The serotonin (5-HT) 2A/C agonist 2,5-Dimethoxy-4-iodoamphetamine (DOI) was tested only in the male rats, which revealed no significant effect of dose, vendor, or interaction on either %PPI or startle amplitude, up to doses that produced adverse effects (lethargy, splayed posture). DOI was therefore terminated from the study before testing was explored in females, as the risk of adverse effects and low likelihood of useful effects was not deemed ethically sound.

**Supplemental Figure S2. Vehicle levels of PPI and startle amplitude**

****Percent PPI (A) and startle amplitude during startle (“pulse”) alone trials (B) over vehicle tests. Abcissae: vehicle test sequence (“apo0”: vehicle in apomorphine dose-effect sequence, “scop0”: vehicle in scopolamine dose-effect sequence, “diz0”: vehicle in dizocilpine dose-effect sequence. Ordinates: percent prepulse inhibition of acoustic startle (A) or amplitude of acoustic startle (B, arbitrary units). Open symbols denote Charles River Sprague-Dawley rats and closed symbols denote Envigo Sprague-Dawley rats. *p<0.05 (main vendor effect).

**Supplemental Table S1**.

Three-way ANOVA with pretreatment dose (repeated), vendor, and sex

| *Apomorphine PPI* |  | |
| --- | --- | --- |
| Vendor | F(1,34)=29.7, *p*<0.0001 | |
| Sex | F(1,34)=1.46, *p*=0.23 | |
| Vendor by sex interaction | F(1,34)=0.91, *p*=0.34 | |
| Apomorphine dose | F(3,78)=7.64, *p*=0.0006 | |
| Apomorphine by vendor interaction | F(3,78)=0.63, *p*=0.59 | |
| Apomorphine by sex interaction | F(3,78)=0.27, *p*=0.84 | |
| 3-way interaction | F(3,78)=0.11, *p*=0.95 | |
| *Scopolamine PPI* |  | |
| Vendor | F(1,25)=5.43, *p*=0.02 | |
| Sex | F(1,25)=6.96, *p*=0.01 | |
| Vendor by sex interaction | F(1,25)=2.81, *p*=0.10 | |
| Scopolamine dose | F(3,75)=1.55, *p*=0.23 | |
| Scopolamine by vendor interaction | F(3,75)=1.72, *p*=0.17 | |
| Scopolamine by sex interaction | F(3,75)=1.38, *p*=0.26 | |
| 3-way interaction | F(3,75)=2.68, *p*=0.053 | |
| *Dizocilpine PPI* | |  |
| Vendor | | F(1,25)=1.64, *p*=0.20 |
| Sex | | F(1,25)=6.02, *p*=0.02 |
| Vendor by sex interaction | | F(1,25)=0.18, *p*=0.67 |
| Dizocilpine dose | | F(1,68)=26.6, *p*<0.0001 |
| Dizocilpine by vendor interaction | | F(1,68)=3.49, *p*=0.03 |
| Dizocilpine by sex interaction | | F(1,68)=3.29, *p*=0.04 |
| 3-way interaction | | F(1,68)=1.58, *p*=0.22 |
| *Apomorphine startle* | |  |
| Vendor | | F(1,34)=45.1, *p*<0.0001 |
| Sex | | F(1,34)=13.1, *p*=0.0005 |
| Vendor by sex interaction | | F(1,34)=0.06, *p*=0.81 |
| Apomorphine dose | | F(1,78)=0.38, *p*=0.77 |
| Apomorphine by vendor interaction | | F(1,78)=1.93, *p*=0.13 |
| Apomorphine by sex interaction | | F(1,78)=2.19, *p*=0.10 |
| 3-way interaction | | F(1,78)=0.79, *p*=0.50 |
| *Scopolamine startle* | |  |
| Vendor | | F(1,25)=31.9, *p*<0.0001 |
| Sex | | F(1,25)=8.59, *p*=0.005 |
| Vendor by sex interaction | | F(1,25)=2.83, *p*=0.10 |
| Scopolamine dose | | F(1,75)=0.94, *p*=0.44 |
| Scopolamine by vendor interaction | | F(1,75)=0.05, *p*=0.99 |
| Scopolamine by sex interaction | | F(1,75)=2.48, *p*=0.07 |
| 3-way interaction | | F(1,68)=0.01, *p*>0.99 |
| *Dizocilpine startle* | |  |
| Vendor | | F(1,25)=108, *p*<0.0001 |
| Sex | | F(1,25)=0.46, *p*=0.50 |
| Vendor by sex interaction | | F(1,25)=5.25, *p*=0.03 |
| Dizocilpine dose | | F(1,68)=4.74, *p*=0.02 |
| Dizocilpine by vendor interaction | | F(1,68)=9.39, *p*=0.0002 |
| Dizocilpine by sex interaction | | F(1,68)=0.06, *p*=0.96 |
| 3-way interaction | | F(1,68)=1.05, *p*=0.34 |
